# Por que a Cardiologia Não Pode mais Ignorar a Saúde Sexual?

**DOI:** 10.36660/abc.20250326

**Published:** 2025-06-18

**Authors:** Ricardo Stein, Filipe Ferrari

**Affiliations:** 1 Universidade Federal do Rio Grande do Sul Faculdade de Medicina Porto Alegre RS Brasil Programa de Pós-Graduação em Cardiologia e Ciências Cardiovasculares, Faculdade de Medicina, Universidade Federal do Rio Grande do Sul, Porto Alegre, RS – Brasil; 2 Hospital de Clínicas de Porto Alegre Grupo de Pesquisa em Cardiologia do Exercício Porto Alegre RS Brasil Grupo de Pesquisa em Cardiologia do Exercício (CardioEx), Hospital de Clínicas de Porto Alegre, Porto Alegre, RS – Brasil; 3 Universidade Federal do Rio Grande do Sul Departamento de Medicina Interna Porto Alegre RS Brasil Departamento de Medicina Interna, Universidade Federal do Rio Grande do Sul, Porto Alegre, RS – Brasil

**Keywords:** Comportamento Sexual, Doenças Cardiovasculares, Aconselhamento

## Introdução

Em uma era de diagnósticos sofisticados e terapias personalizadas, um aspecto do atendimento ao paciente permanece rotineiramente negligenciado: a saúde sexual. A atividade sexual (AS) é mais do que uma fonte de prazer; é também um marcador de vitalidade, intimidade e resiliência emocional.^1–3^ Para indivíduos com doença cardiovascular (DCV), o desejo de retomar a AS frequentemente sinaliza não apenas a recuperação física, mas também a cura emocional e a renovação da autoconfiança. Por outro lado, incentivamos os pacientes a caminhar, a se alimentar melhor e a tomar seus medicamentos. No entanto, quando se trata de sexo, o silêncio prevalece.

Embora a AS imponha demandas cardiovasculares comparáveis à atividade física moderada e seja geralmente considerada segura para a maioria dos pacientes com DCV estável,^4^ muitos permanecem incertos sobre quando ou como retomar a vida sexual após infarto do miocárdio (IM),^5^ cirurgias de revascularização do miocárdio^6^ e insuficiência cardíaca.^7^ Eles ficam com perguntas sem resposta porque o tópico é frequentemente recebido com desconforto ou garantias vagas. Estudos têm mostrado consistentemente que os pacientes querem – e precisam – de orientação clara sobre o funcionamento sexual. No entanto, tabus culturais, constrangimentos e falta de treinamento profissional continuam a silenciar essa conversa em ambientes clínicos.^8,9^ Em um estudo, apenas 16% dos cardiologistas discutiam rotineiramente a função sexual, enquanto 70% raramente ou nunca ofereciam orientação após o IM.^9^ As barreiras mais comumente citadas incluíam restrições de tempo, falta de privacidade e treinamento insuficiente.

Este é um problema generalizado. Na Austrália, menos de 25% dos profissionais de saúde abordam rotineiramente a saúde sexual de pacientes pós-IM, apesar de reconhecerem sua importância.^10^ No Irã, a maioria dos cardiologistas reconheceu a relevância do tema, mas admitiu sentir-se despreparada e raramente iniciar essas conversas.^11^ O padrão é claro: reconhecimento sem ação. Além disso, os pacientes estão pagando o preço.

### Risco cardiovascular e atividade sexual

Então, quais são os riscos reais? A morte súbita cardíaca durante a AS é rara, representando menos de 2% das mortes relacionadas ao exercício, e o risco geral de IM durante o sexo permanece baixo.^2^ Um estudo britânico com 6.847 casos de morte súbita cardíaca descobriu que apenas 0,2% ocorreram durante ou dentro de uma hora após a AS.^12^ Além disso, uma meta-análise mostrou que, embora a AS episódica possa aumentar ligeiramente o risco de IM ou morte súbita cardíaca, o risco absoluto é mínimo: apenas 2 a 3 infartos do miocárdio adicionais e 1 morte súbita a cada 10.000 pessoas-ano para cada hora extra de AS por semana.^13^ É importante ressaltar que indivíduos regularmente ativos – especialmente mulheres – enfrentam um risco ainda menor.

### Considerações farmacológicas e fisiológicas

A saúde sexual não é apenas uma questão de psicologia ou relacionamentos – ela está profundamente ligada à fisiologia cardiovascular e aos medicamentos que prescrevemos. Muitos pacientes apresentam discretamente redução da libido ou disfunção erétil, frequentemente atribuídas a betabloqueadores, diuréticos ou anti-hipertensivos.^14–17^ Outros lutam com as consequências vasculares da insuficiência cardíaca, disfunção endotelial ou fadiga crônica, que podem corroer a confiança e a intimidade.

Betabloqueadores, por exemplo, têm sido associados à disfunção erétil há muito tempo, particularmente agentes mais antigos como o tartarato de metoprolol. Em contraste, o nebivolol parece oferecer um perfil mais favorável.^14^ Da mesma forma, antagonistas da aldosterona, como espironolactona e eplerenona, podem prejudicar a função sexual devido aos efeitos antiandrogênicos e à supressão da secreção de gonadotrofinas.^15^ Embora frequentemente ignorados, esses efeitos diminuem significativamente a qualidade de vida. Notavelmente, essas não são preocupações periféricas. Elas moldam a maneira como os pacientes convivem com sua doença e como se sentem em relação a si mesmos. Quando um paciente relata fadiga, pode também estar de luto pela perda da vida sexual. Quando prescrevemos terapias que prolongam a vida, devemos também considerar seu impacto em experiências que melhoram a vida, como intimidade e conexão. As escolhas farmacológicas não devem ser feitas isoladamente. Sempre que possível, devemos selecionar regimes que equilibrem eficácia com bem-estar sexual. Acima de tudo, os pacientes devem se sentir seguros ao expressar essas preocupações. Essa segurança começa conosco.

### O papel do cardiologista no aconselhamento em saúde sexual

Cardiologistas estão em uma posição única para abordar a saúde sexual, mas muitas vezes permanecem em silêncio. Discutir proativamente esse tópico durante as consultas deve se tornar rotina, não exceção. Uma conversa breve e empática pode normalizar o problema, reduzir o estigma e abrir caminho para um diálogo mais profundo.

**Figura 1:**
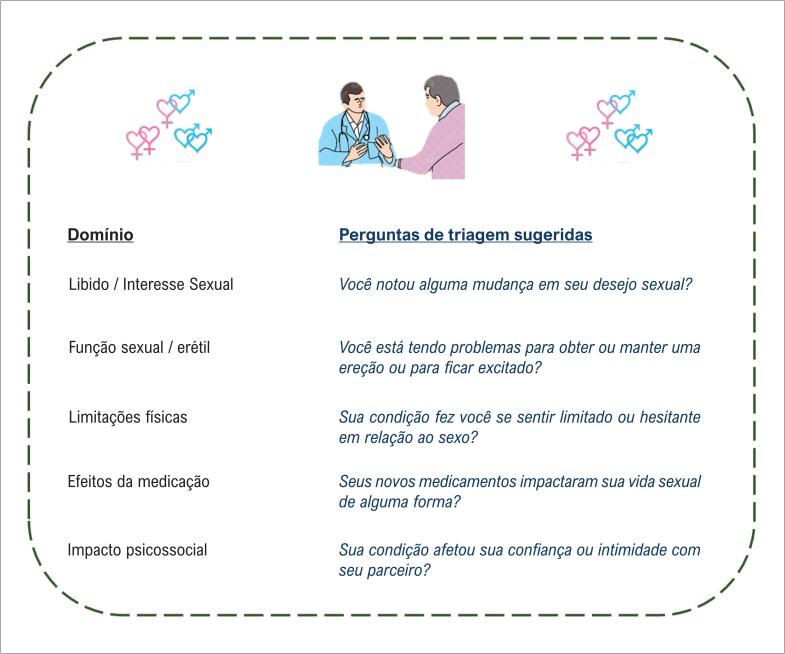
Perguntas breves de triagem para saúde sexual em pacientes cardiovasculares. Essas perguntas são destinadas a uma triagem breve e empática e podem orientar encaminhamentos para especialistas apropriados, quando necessário.

Ferramentas e questionários simples podem ajudar a identificar a disfunção sexual e suas possíveis ligações com doenças cardiovasculares ou efeitos colaterais de medicamentos. A Figura 1 apresenta um exemplo prático de como os médicos podem iniciar a conversa de forma eficiente e empática. Incluir essas ferramentas em avaliações de rotina, assim como fazemos para depressão ou atividade física, sinaliza aos pacientes que sua saúde sexual é importante.

Os cardiologistas não precisam fazer isso sozinhos. O atendimento ideal geralmente requer uma abordagem multidisciplinar, envolvendo a colaboração de urologistas, psicólogos, terapeutas sexuais e médicos de atenção primária. Assim como encaminhamos pacientes para o manejo da apneia do sono ou do diabetes, devemos nos sentir igualmente confiantes ao encaminhar para problemas de saúde sexual.

Sexo não é um luxo; é um aspecto fundamental da experiência humana. Além disso, como responsáveis pelo bem-estar cardiovascular e emocional dos nossos pacientes, devemos tratá-los como tais.

## Conclusões

Evitar conversas sobre AS faz mais mal do que bem. Priva os pacientes da segurança, orientação e apoio de que precisam para retomar uma parte essencial da vida, e mina a própria base do cuidado holístico.

É hora de a cardiologia tirar a sexualidade das sombras. Precisamos capacitar os profissionais para abordá-la de forma aberta, precisa e compassiva, assim como faríamos com qualquer outro aspecto do risco cardiovascular ou da recuperação. Se estivermos verdadeiramente comprometidos com o cuidado centrado no paciente, a saúde sexual não deve mais ser uma preocupação secundária.
