# Supplementary material for: Por que a Cardiologia Não Pode mais Ignorar a Saúde Sexual?
Source: Arq Bras Cardiol. 2025 Jun 18;122(6):e20250326. [Article in Portuguese] doi: 10.36660/abc.20250326 (PMC12212391; doi:10.36660/abc.20250326)
Supplement: Translation

# Translation: Why Cardiology Can No Longer Ignore Sexual Health?

**DOI:** 10.36660/abc.20250326i

**Authors:** Ricardo Stein, Filipe Ferrari

**Affiliations:** 1 Porto Alegre RS Brazil Programa de Pós-Graduação em Cardiologia e Ciências Cardiovasculares, Faculdade de Medicina, Universidade Federal do Rio Grande do Sul, Porto Alegre, RS – Brazil; 2 Porto Alegre RS Brazil Grupo de Pesquisa em Cardiologia do Exercício (CardioEx), Hospital de Clínicas de Porto Alegre, Porto Alegre, RS – Brazil; 3 Porto Alegre RS Brazil Departamento de Medicina Interna, Universidade Federal do Rio Grande do Sul, Porto Alegre, RS – Brazil

**Keywords:** Sexual Behavior, Cardiovascular Diseases, Counseling

## Introduction

In an era of sophisticated diagnostics and personalized therapies, one aspect of patient care remains routinely overlooked: sexual health. Sexual activity (SA) is more than a source of pleasure; it is also a marker of vitality, intimacy, and emotional resilience.^1–3^ For individuals with cardiovascular disease (CVD), the desire to resume SA often signals not only physical recovery but also emotional healing and renewed self-confidence. On the other hand, we encourage patients to walk, to eat better, and to take their medications. Nevertheless, when it comes to sex, silence prevails.

Although SA imposes cardiovascular demands comparable to moderate physical activity and is generally considered safe for most patients with stable CVD,^4^ many remain uncertain about when or how to resume sexual life after myocardial infarction (MI),^5^ coronary artery bypass graft surgery,^6^ and heart failure.^7^ They are left with unanswered questions because the topic is often met with discomfort or vague reassurances. Studies have consistently shown that patients want – and need – clear guidance on sexual functioning. However, cultural taboos, embarrassment, and lack of professional training continue to silence this conversation in clinical settings.^8,9^ In one study, only 16% of cardiologists routinely discussed sexual function, while 70% rarely or never offered guidance after MI.^9^ The most commonly cited barriers included time constraints, lack of privacy, and insufficient training.

This is a widespread issue. In Australia, fewer than 25% of healthcare professionals routinely address sexual health with post-MI patients, despite recognizing its importance.^10^ In Iran, most cardiologists acknowledged the relevance of the topic but admitted feeling unprepared and rarely initiated these conversations.^11^ The pattern is clear: recognition without action. Furthermore, patients are paying the price.

### Cardiovascular risk and sexual activity

So what are the real risks? Sudden cardiac death during SA is rare, accounting for less than 2% of exercise-related deaths, and the overall risk of MI during sex remains low.^2^ An UK study of 6,847 sudden cardiac death cases found that only 0.2% occurred during or within one hour of SA.^12^ Furthermore, a meta-analysis showed that although episodic SA may slightly increase the risk of MI or sudden cardiac death, the absolute risk is minimal: only 2-3 additional MIs and 1 sudden death per 10,000 person-years for each extra hour of SA per week.^13^ Importantly, regularly active individuals – especially women – face an even lower risk.

### Pharmacological and physiological considerations

Sexual health is not merely a matter of psychology or relationships – it is deeply connected to cardiovascular physiology and the medications we prescribe. Many patients quietly experience reduced libido or erectile dysfunction, often attributed to beta-blockers, diuretics, or anti-hypertensives.^14–17^ Others struggle with the vascular consequences of heart failure, endothelial dysfunction, or chronic fatigue, all of which can erode confidence and intimacy.

Beta-blockers, for instance, have long been associated with erectile dysfunction, particularly older agents like metoprolol tartrate. In contrast, nebivolol appears to offer a more favorable profile.^14^ Similarly, aldosterone antagonists such as spironolactone and eplerenone may impair sexual function due to anti-androgenic effects and suppression of gonadotropin secretion.^15^ Though often overlooked, these effects significantly diminish quality of life. Notably, these are not peripheral concerns. They shape how patients live with their disease and how they feel about themselves. When a patient reports fatigue, they may also be mourning a lost sex life. When we prescribe life-prolonging therapy, we must also consider its impact on life-enhancing experiences, such as intimacy and connection. Pharmacological choices should not be made in isolation. Whenever possible, we should select regimens that balance efficacy with sexual well-being. Above all, patients should feel safe raising these concerns. That safety begins with us.

### The role of the cardiologist in sexual health counseling

Cardiologists are uniquely positioned to address sexual health, yet too often remain silent. Proactively discussing this topic during consultations should become routine, not the exception. A brief, empathetic conversation can normalize the issue, reduce stigma, and open the door for deeper dialogue.

Simple tools and questionnaires can help identify sexual dysfunction and its possible links to CVD or medication side effects. Figure 1 provides a practical example of how clinicians can efficiently and empathetically initiate the conversation. Including such tools in routine assessments, just as we do for depression or physical activity, signals to patients that their sexual health matters.

**Figure 1:** Brief Screening Questions for Sexual Health in Cardiovascular Patients. These questions are intended for brief, empathetic screening and may guide referrals to appropriate specialists when needed.

Cardiologists do not have to do this alone. Optimal care often requires a multidisciplinary approach, involving collaboration with urologists, psychologists, sex therapists, and primary care providers. Just as we refer patients for sleep apnea or diabetes management, we should feel equally confident referring for sexual health concerns.

Sex is not a luxury; it is a fundamental aspect of the human experience. Moreover, as stewards of our patients’ cardiovascular and emotional well-being, we must treat them as such.

## Conclusions

Avoiding conversations about SA does more harm than good. It deprives patients of the reassurance, guidance, and support they need to reclaim an essential part of life – and undermines the very foundation of holistic care.

It is time for cardiology to bring sexuality out of the shadows. We must empower professionals to address it openly, accurately, and compassionately, just as we would any other aspect of cardiovascular risk or recovery. If we are truly committed to patient-centered care, sexual health must no longer be an afterthought.
